# Plasma levels of platelet-enriched microRNAs change during antiplatelet therapy in healthy subjects

**DOI:** 10.3389/fphar.2022.1078722

**Published:** 2022-12-12

**Authors:** Teresa L. Krammer, Marietta Kollars, Paul A. Kyrle, Matthias Hackl, Sabine Eichinger, Ludwig Traby

**Affiliations:** ^1^ TAmiRNA GmbH, Vienna, Austria; ^2^ Department of Medicine I, Medical University of Vienna, Vienna, Austria

**Keywords:** platelet-enriched microRNA, thrombomiR, biomarker, antiplatelet therapy, extracellular vesicle, procoagulant vesicle, clopidogrel, ticagrelor

## Abstract

Platelets are the main effectors of primary hemostasis but also cause thrombosis in pathological conditions. Antiplatelet drugs are the cornerstone for the prevention of adverse cardiovascular events. Monitoring the extent of platelet inhibition is essential. Currently available platelet function tests come with constraints, limiting use in antiplatelet drug development as well as in clinical routine. With this study, we aim to investigate whether plasma miRNAs might be suitable biomarkers for monitoring antiplatelet treatment. Platelet-poor plasma was obtained from a trial including 87 healthy male volunteers that either received ticagrelor (*n* = 44) or clopidogrel (*n* = 43). Blood was collected before drug intake and after 2 h, 6 h, and 24 h. We measured a panel of 11 platelet-enriched miRNAs (thrombomiRs) by RT-qPCR and selected four biomarker candidates (i.e., miR-223-3p, miR-150-5p, miR-126-3p, miR-24-3p). To further characterize those miRNAs, we performed correlation analyses with the number of extracellular vesicles and clotting time dependent on procoagulant vesicles (PPL assay). We show that platelet-enriched miRNAs in the circulation are significantly reduced upon P2Y12-mediated platelet inhibition. This effect occurred fast, reaching its peak after 2 h. Additionally, we demonstrate that higher baseline levels of thrombomiRs are linked to a stronger reduction upon antiplatelet therapy. Finally, we show that miRNAs from our panel might be the cargo of platelet-derived and procoagulant vesicles. In conclusion, we provide evidence that thrombomiR levels change within 2 h after pharmacological platelet inhibition and circulate the body within platelet-derived and procoagulant extracellular vesicles, rendering them potential biomarker candidates for the assessment of *in vivo* platelet function.

## 1 Introduction

Platelets are small cells without a nucleus that are shed from polyploid megakaryocytes in the bone marrow. As main effector cells of primary hemostasis, platelets are essential to minimize blood loss upon injury. However, deregulated platelet function is causally connected to cardiovascular diseases (CVDs) such as stroke or myocardial infarction. Patients suffering from CVDs might have elevated levels of activated platelets and platelet-derived extracellular vesicles (PdEVs) (Amraotkar et al., 2017; Zaldivia et al., 2017). To prevent adverse cardiovascular events, antiplatelet agents are successfully used in clinical practice. In the course of pharmacological platelet inhibition, a balance between high on-treatment platelet reactivity and severe bleeding must be maintained. Therefore, monitoring the extent of platelet inhibition would be beneficial. Yet, presently available platelet function tests (PFTs) have considerable limitations, including lack of standardization, complicating the interpretation of results and hampering the comparison with additional tests. Moreover, standard PFTs require fresh blood and usually only allow the assessment of one activation pathway at a time. The use of PFTs in a personalized medicine fashion to guide antiplatelet treatment remains a matter of controversy. A novel biomarker to detect patients with excessive or inadequate response to platelet inhibition would be advantageous. Cell-free microRNAs (miRNAs) circulating in peripheral blood have been suggested as promising biomarker candidates in the context of CVDs (Zampetaki et al., 2012a; Zhou et al., 2018). MiRNAs are approximately 22 nt long non-coding RNAs that repress messenger RNA translation and thereby fine-tune protein expression (Baek et al., 2008; Bartel, 2018). Extracellular miRNAs in the blood are protected from degradation as they are either bound to proteins (Argonaute, lipoproteins) or complexed with EVs (Valadi et al., 2007; Hunter et al., 2008; Arroyo et al., 2011; Vickers et al., 2011). Up to 750 different miRNA species constitute the dynamic platelet miRNome (Bray et al., 2013; Sunderland et al., 2017). Landry et al. (2009) report that one of the most abundant platelet miRNAs, miR-223, regulates the expression of the P2Y12 receptor, influencing platelet reactivity. Nevertheless, for the majority of miRNAs detected in platelets, specific functions remain to be uncovered. Platelets strongly contribute to the pool of circulatory miRNAs and are capable of processing precursor miRNAs (pre-miRNAs), despite their small size and lack of a nucleus (Landry et al., 2009). Over one decade ago, intracellular platelet miRNA levels were linked to platelet reactivity, paving the way for additional research into the utility of miRNAs as biomarkers of platelet function (Nagalla et al., 2011). Several studies have investigated whether circulating levels of platelet-enriched miRNAs are responsive to antiplatelet treatment (de Boer et al., 2013; Willeit et al., 2013; Carino et al., 2016; Jäger et al., 2019; Parker et al., 2020). Willeit et al. (2013) and colleagues report reduced levels of platelet-enriched miRNAs in the plasma of healthy volunteers upon escalating doses of prasugrel in combination with aspirin. This finding was validated in a cohort of patients suffering from symptomatic carotid atherosclerosis after onset of dual antiplatelet therapy (DAPT) (Willeit et al., 2013). It has been proposed that activated platelets release miRNAs primarily incorporated within EVs (Diehl et al., 2012; Laffont et al., 2013). EVs are submicron vesicles emitted from membranes of a broad variety of cells. A substantial amount of EVs in plasma carry surface markers indicating platelet or megakaryocyte origin (Flaumenhaft et al., 2009; Rank et al., 2010; Arraud et al., 2014). While the miRNA cargo of PdEVs is insufficiently understood (Provost, 2017), their protein content (Capriotti et al., 2013; Milioli et al., 2015; Suades et al., 2022) and procoagulant activity (Sinauridze et al., 2007; Tripisciano et al., 2017) have been characterized. Evidence gathered by several studies suggests that some platelet miRNAs in plasma are promising biomarker candidates to monitor *in vivo* platelet function. However, there is currently no comprehensive study demonstrating the dynamic effects of platelet inhibition on circulatory miRNA levels in a sufficiently large cohort. To this end, we performed RT-qPCR in platelet-poor plasma (PPP) of healthy male volunteers either receiving ticagrelor or clopidogrel. We i.) measured therapy-induced alterations in miRNA levels (thrombomiR panel) over several time points; ii.) examined the connection between high baseline levels and the magnitude of miRNA reduction; and iii.) performed correlation analyses of platelet-derived, endothelial cell-derived, and procoagulant EVs with our miRNA panel to elucidate potential carriers of platelet-enriched miRNAs.

## 2 Materials and methods

### 2.1 Study population and design

Between November 2011 and December 2013, we conducted a randomized, parallel-group, double-blind, placebo-controlled trial at the Department of Medicine I and Clinical Pharmacology of the Medical University of Vienna (Traby et al., 2021, 2020). The trial is listed on clinicaltrials.gov (NCT02120092) and the European clinical trials database (EudraCT 2010-019643-19). The study has been approved by the ethics committee of the Medical University of Vienna, Austria. The trial was performed in healthy male volunteers and was carried out in two parts. A detailed description of the study population and the design has been published (Traby et al., 2021, Traby et al., 2020). Briefly, in part I, subjects received a loading dose of 600 mg clopidogrel (Plavix; Sanofi Pharma Bristol-Myers Squibb) together with either 100 mg aspirin or placebo on day 1 and a maintenance dose of 150 mg clopidogrel (and 100 mg aspirin or placebo) from days 2–7. In part II, subjects received a single dose of 180 mg ticagrelor (Brilique/Brilinta; AstraZeneca; Södertälje, Sweden) with either 300 mg aspirin or placebo.

### 2.2 Blood collection from healthy subjects and preparation of plasma

Venous blood was drawn from the antecubital vein using either an 18-gauge catheter or a 21-gauge needle into tubes containing 3.8% sodium citrate (Vacuette; Greiner BioOne, Kremsmünster, Austria). Blood was collected before (baseline) and 2 h, 6 h, and 24 h after drug intake. Samples were centrifuged twice at 2,600x g for 15 min at 4°C and the resulting PPP was stored at -80 °C until further processing.

### 2.3 RNA extraction and qPCR analysis

Total RNA was isolated from 200 μl PPP using the Maxwell RSC48 with the miRNA Tissue Kit (Promega). Samples were thawed at room temperature and centrifuged for 5 min at 12,000x g at 4°C to remove cellular debris. Samples were then homogenized with 200 μl homogenization solution (containing thioglycerol) and 200 μl lysis solution (containing 1 μl/sample RNA spike-in). 15 μl Proteinase K were added to each sample followed by 15 min incubation at 37°C and 300 rpm. The “simply RNA tissue” program was used and RNA was eluted in 50 μl nuclease-free water and stored at −80°C until further processing. Reverse transcription and quantitative PCR (qPCR) analysis were performed as previously described (Krammer et al., 2022). In short, for cDNA synthesis, the miRCURY RT kit (Qiagen, Venlo, Netherlands) was used with 2 μl total RNA input. qPCR was performed using the miRCURY SYBR® Green Master Mix with commercial LNA-enhanced miRNA assays (Qiagen) and a final cDNA dilution of 1:100. With this setup, only mature miRNAs are detected. Quality control was performed using synthetic spike-ins (Qiagen) that were added in equimolar amounts to each sample prior to each step of the workflow. qPCRs were performed on a LightCylcer480 II (Roche) with the following settings: 95°C for 2 min, 45 cycles of 95°C for 10 s and 56°C for 60 s. Melting curve analysis was performed using continuous acquisition between 55 and 99°C. Cycle threshold (Cq) values were calculated with the 2nd derivative maximum method (LC480, Roche v1.5.1.62). The hemolysis ratio was calculated as previously described (Blondal et al., 2013) and hemolytic samples were removed from further analysis. RNA (UniSp4), cDNA (cel-miR-39-3p), and PCR (UniSp3) spike-ins were measured in all samples (Supplementary Figure S1). Samples that failed quality control were removed from the data set. Cq values were normalized with the RNA spike-in control as internal standard (Zampetaki and Mayr, 2012b). The following formula was applied to calculate ΔCq values:

$$\Delta Cq=Cq\left(RNA\;spike‐in\right)-Cq\left(target\;miRNA\right)$$

### 2.4 Measurement of platelet parameters

Platelet counts were determined according to hospital routine methods using large scale hematologic analyzers (XN-Series, Sysmex Europe SE, Germany).

### 2.5 Extracellular vesicle characterization using flow cytometry

Vesicle isolation and characterization was performed as previously described (Traby et al., 2018). In short, particles <1 μm (forward scatter) and Annexin V^+^ (side scatter) were counted as EVs. For EV characterization, antibodies against surface molecules were applied. CD41a was used to identify EVs released from platelets and CD105 indicates EVs of endothelial cell origin.

### 2.6 Clotting time dependent on PPL

To measure phospholipid-dependent clotting time (PPL), the STA-Procoag-PPL assay (Stago, Asnières sur Seine, France) was used on a Cobas coagulation analyzer (Roche, Germany) (Traby et al., 2021). Results signify coagulation times [s] in PPP. The more procoagulant phospholipids are present, the shorter is the measured coagulation time.

### 2.7 Statistical analysis

Only non-parametric statistical tests were applied to our data set. The specific statistical tests are elaborated within the caption of each figure. Box plots range from minimum to maximum and display the median. Scatter plots with bars display the median with the 95% confidence interval. Relative expression was determined by linearizing normalized data (∆Cq_UniSp4_) and calculating relative expression as percentage of the median of baseline samples. In case of paired data, donors with missing values were removed to allow pairwise analysis. Correlations were calculated using the Spearman test. Through simple linear regression, a line was fit to the data. *p*-Values < 0.05 were considered statistically significant. Multiple testing adjusted *p*-values are reported as false discovery rate (FDR) for each miRNA. All statistical analyses were performed using GraphPad Prism v9.4.0 (www.graphpad.com).

## 3 Results

### 3.1 Subjects

44 male volunteers were included in the ticagrelor arm and 43 in the clopidogrel arm of the trial. The respective P2Y12 receptor inhibitors were administered either in combination with aspirin (DAPT) or as mono antiplatelet therapy (MAPT) together with placebo. The median age of the subjects was 24 years (25th; 75th percentile: 23; 27) and 25 years (25th; 75th percentile: 23; 27), respectively. Subjects in the individual arms did not differ significantly with regard to platelet counts, hemoglobin, results of global coagulation assays, and fibrinogen levels. None of the volunteers had a severe adverse event.

### 3.2 Addition of aspirin to the P2Y12 receptor inhibitors ticagrelor and clopidogrel does not affect levels of platelet-enriched miRNAs in blood

**FIGURE 1:**
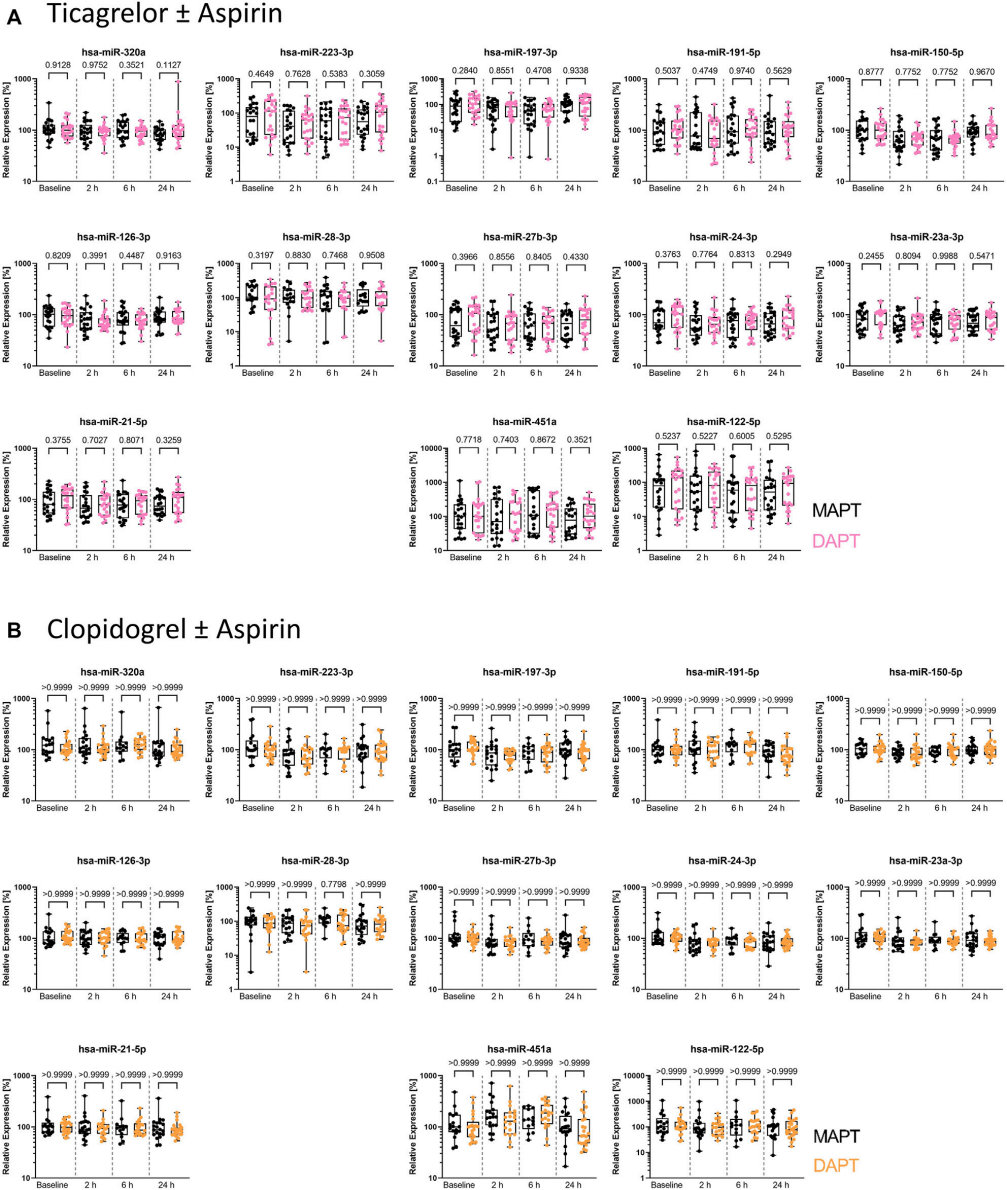
Plasma levels of miRNAs from the thrombomiR panel are not affected by addition of aspirin to P2Y12 inhibitors, warranting combined analysis of MAPT and DAPT data. **(A)** MiRNA levels in subjects treated with ticagrelor and aspirin or placebo (independent donors MAPT = 22; independent donors DAPT = 22). **(B)** MiRNA levels in subjects treated with clopidogrel and aspirin or placebo (independent donors MAPT = 20; independent donors DAPT = 23). Values represent the relative expression of UniSp4 normalized Cq values that were linearized (ΔΔCq, % of the median of baseline samples). Kruskal-Wallis test with Dunn’s multiple comparisons test was calculated. *p*-values <0.05 were considered significant.

We analyzed miRNA levels in PPP of young, healthy donors from both arms of the clinical trial. We utilized the thrombomiR panel, featuring 11 platelet-enriched miRNAs as well as the liver-specific miR-122-5p, serving as negative control. To ensure the quality of the generated data, we added synthetic spike-ins in equimolar amounts before each step of the workflow (RNA isolation, reverse transcription, qPCR) (Supplementary Figure S1). At first, we determined whether addition of aspirin to P2Y12 receptor inhibitors leads to significant differences in miRNA levels. We found that the combination of aspirin with ticagrelor or clopidogrel did not significantly change thrombomiR levels (Figures 1A, B). We therefore decided to combine MAPT and DAPT data to focus on the effect of platelet inhibition on circulating miRNA levels. All miRNAs from the thrombomiR panel were clearly detectable at baseline as the selected miRNAs are platelet-enriched but not platelet-specific (other cell types contribute to basal levels). Of note, we observed a strong inter-individual variability at baseline, reaching up to 6 Cq values in miR-223-3p (ticagrelor cohort), signifying a 64-fold difference. In order to further investigate potential sources of variation, we correlated thrombomiR levels with platelet counts at baseline but did not observe a strong association (Supplementary Figures S2A, B).

### 3.3 Circulating thrombomiRs are significantly reduced upon initiation of platelet inhibition

Seven out of 11 thrombomiRs were decreased upon antiplatelet therapy in both cohorts. MiR-21-5p was significantly decreased in the ticagrelor arm but not upon clopidogrel treatment. MiR-320a, miR-191-5p, and miR-28-3p were not reduced upon pharmacological platelet inhibition (Supplementary Figures S3A, B). We selected four top candidates from our panel considering effect size and redundancy of information (i.e., only one miRNA from the miR-23–27∼24 clusters): miR-223-3p, miR-150-5p, miR-126-3p, and miR-24-3p. All of the selected miRNAs (miR-223-3p, miR-150-5p, miR-126-3p, miR-24-3p) were significantly decreased upon treatment with ticagrelor or clopidogrel. The effect of antiplatelet therapy on platelet-enriched miRNA levels in the circulation occurred fast, peaking 2 h after therapy onset.

#### 3.3.1 Effect of ticagrelor on circulating miRNA levels

**FIGURE 2:**
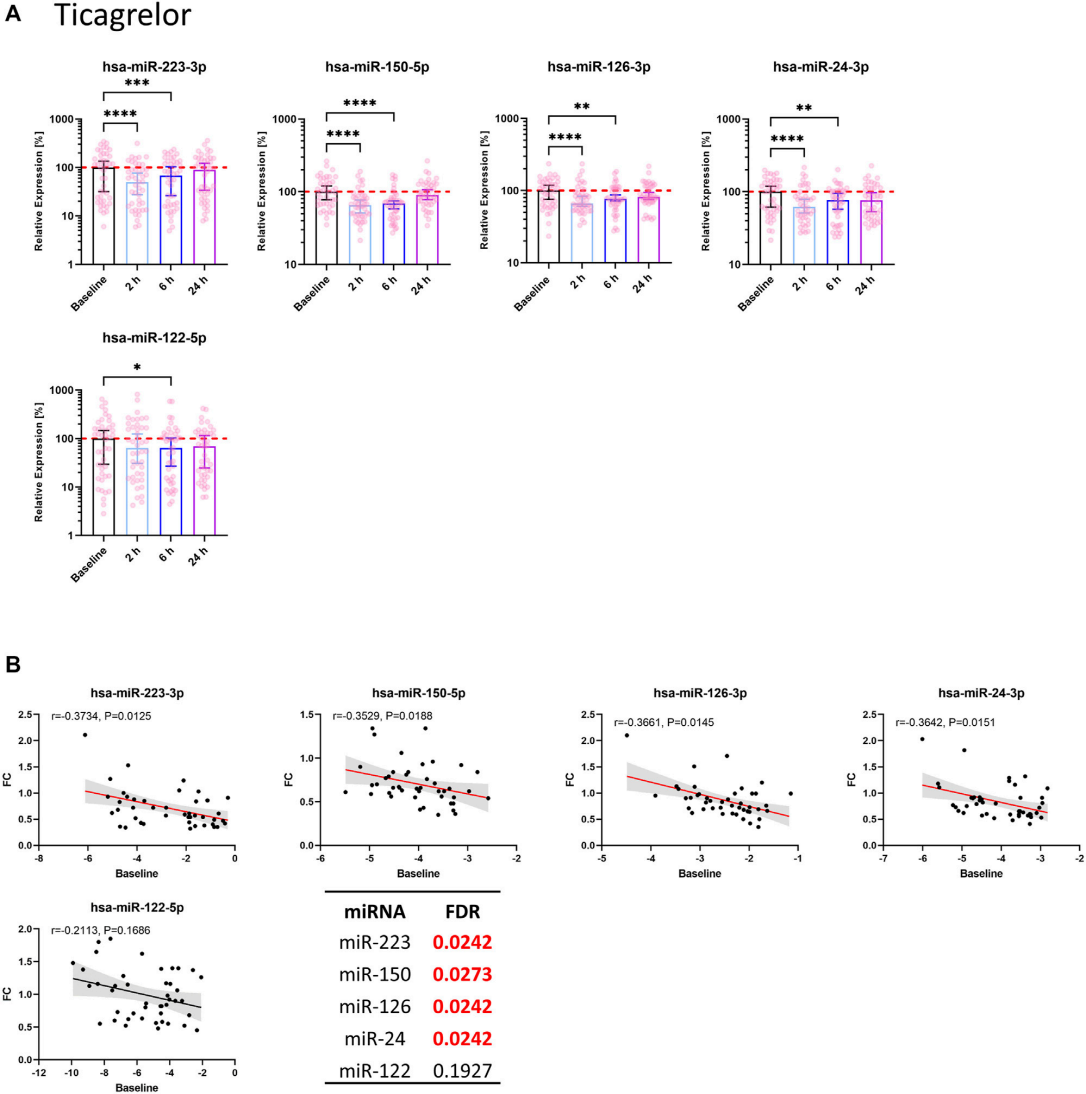
Circulating levels of miRNAs from the thrombomiR panel are significantly reduced upon ticagrelor-mediated inhibition of platelet function. **(A)** MiRNA levels in subjects treated with ticagrelor. Values represent the relative expression of UniSp4 normalized Cq values that were linearized (ΔΔCq, % of the median of baseline samples). Friedman test with Dunn’s multiple comparisons test was calculated. Donors with missing values were removed from statistical analysis but not from depiction in graphs. **(B)** Baseline miRNA levels were correlated with the FC of the decrease 2 h after onset of ticagrelor treatment. Correlations were calculated using the Spearman test, reporting the Spearman rank coefficient (r) and unadjusted *p*-value (P). A line was fit to the data applying simple linear regression. Multiple testing adjusted *p*-values are reported as FDR for each miRNA. FDR-values <0.05 were considered statistically significant.

Ticagrelor treatment significantly reduced the levels of miR-223-3p, miR-150-5p, miR-126-3p, and miR-24-3p (Figure 2A). MiR-223-3p was reduced to around 50% of the median of baseline samples 2 h after therapy start (Table 1A). This reduction also occurred in miR-150-5p (∼35%; ≙ decrease to 65% of baseline), miR-126-3p (∼34%), and miR-24-3p (∼38%). After 6 h, the four selected miRNAs were still significantly decreased compared to the median of the baseline. There was a slight but significant reduction of the liver-specific miR-122-5p after 6 h. After 24 h, the chosen miRNAs rebounded to baseline levels and were not significantly reduced anymore. Besides the four top candidates, miR-197-3p, miR-27b-3p, miR-23a-3p, and miR-21-5p were significantly decreased upon ticagrelor treatment as well (Supplementary Figure S3A). Next, we correlated baseline levels with the fold reduction after 2 h to investigate whether the variability at baseline is linked to the magnitude of platelet-enriched miRNA reduction upon platelet inhibition. Baseline levels of miR-223-3p, miR-150-5p, miR-126-3p, and miR-24-3p were significantly correlated with the extent of reduction of circulating miRNA levels 2 h after ticagrelor intake, indicating that donors with high baseline levels undergo a more pronounced reduction upon ticagrelor treatment (Figure 2B). The fold change (FC) of the negative control, miR-122-5p, was not significantly correlated with baseline levels. This effect was further examined by separately analyzing subjects with particularly high miRNA levels and subjects with especially low miRNA levels. We observed a decrease of on average 46% after 2 h in the 10 volunteers with highest miR-223-3p baseline levels, compared to on average 21% reduction in donors with the lowest baseline levels (Table 2A). The same effect occurred in all thrombomiRs from our selection and was most pronounced in miR-126 with no decrease (on average) upon platelet inhibition in donors with low baseline levels and ∼30% decrease in subjects with high miRNA levels.

**TABLE 1:** Effects of ticagrelor (A) and clopidogrel (B) treatment on platelet-enriched miRNA levels in plasma. Median values after 2 h, 6 h, and 24 h are in comparison to the median of the baseline. Donors with missing values were removed from statistical analysis (Friedman test with Dunn’s multiple comparisons test).

	hsa-miR-223-3p	hsa-miR-150-5p	hsa-miR-126-3p	hsa-miR-24-3p	hsa-miR-122-5p
A Ticagrelor					
Median 2 h [%] + 95% CI	50.4 (27–77)	65.3 (51–77)	66.5 (61–84)	61.8 (51–79)	63.5 (31–124)
Δmedian 2 h-BL [percentage points]	−49.7	−34.7	−33.6	−38.2	−36.6
*p*-value	<0.0001	<0.0001	<0.0001	<0.0001	>0.9999
Median 6 h [%] + 95% CI	68.5 (26–104)	68.8 (58–75)	76.9 (72–87)	76.9 (57–95)	64.2 (27–103)
Δmedian 6 h-BL [percentage points]	−31.6	−31.2	−23.2	−23.1	−35.9
*p*-value	0.0008	<0.0001	0.0038	0.0051	0.0247
Median 24 h [%] + 95% CI	88.8 (34–123)	89.8 (78–106)	81.8 (75–95)	76.2 (53–97)	69.3 (25–115)
Δmedian 24 h-BL [percentage points]	−11.3	−10.2	−18.2	−23.8	−30.8
*p*-value	>0.9999	0.3798	0.1570	0.3500	0.0774
B Clopidogrel					
Median 2 h [%] + 95% CI 2 h	80.4 (60–98)	83.2 (76–102)	98.6 (87–113)	77.9 (71–91)	83.2 (64–124)
Δmedian 2 h-BL [percentage points]	−19.6	−16.8	−1.4	−22.1	−16.8
*p*-value	<0.0001	0.0094	0.0157	0.0003	0.047
Median 6 h [%] + 95% CI 6 h	102.8 (85–105)	94.3 (78–110)	98.3 (86–117)	88.0 (69–99)	108.6 (68–165)
Δmedian 6 h-BL [percentage points]	2.8	−5.7	−1.7	−12.0	8.6
*p*-value	0.0055	0.0951	0.0046	0.0026	0.2035
Median 24 h [%] + 95% CI 24 h	91.1 (73–119)	98.3 (87–113)	97.3 (85–116)	81.2 (72–91)	97.6 (64–119)
Δmedian 24 h-BL [percentage points]	−8.9	−1.7	−2.7	−18.8	−2.4
*p*-value	0.6502	>0.9999	0.0255	0.0133	0.358

**TABLE 2:** Comparison of the relative change (2 h vs. baseline) between the donors with the highest and lowest baseline levels: (A) Ticagrelor treatment; (B) clopidogrel treatment. Values below 100% indicate a decrease of the respective miRNA 2 h after onset of platelet inhibition.

	miR-223 (%)	miR-150 (%)	miR-126 (%)	miR-24 (%)	miR-122 (%)
A Ticagrelor					
Average change observed at 2 h in the 10 donors with the lowest baseline levels	79.4	79.3	103.7	97.3	117.8
Average change observed at 2 h in the 10 donors with the highest baseline levels	53.6	56.0	70.2	65.6	92.7
Overall average	64.0	67.1	81.4	78.1	92.2
B Clopidogrel					
Average change observed at 2 h in the 10 donors with the lowest baseline levels	81.4	91.8	91.4	86.1	89.9
Average change observed at 2 h in the 10 donors with the highest baseline levels	57.8	73.3	74.5	59.7	74.6
Overall average	71.3	82.6	88.2	74.7	81.0

#### 3.3.2 Effect of clopidogrel on circulating miRNA levels

**FIGURE 3:**
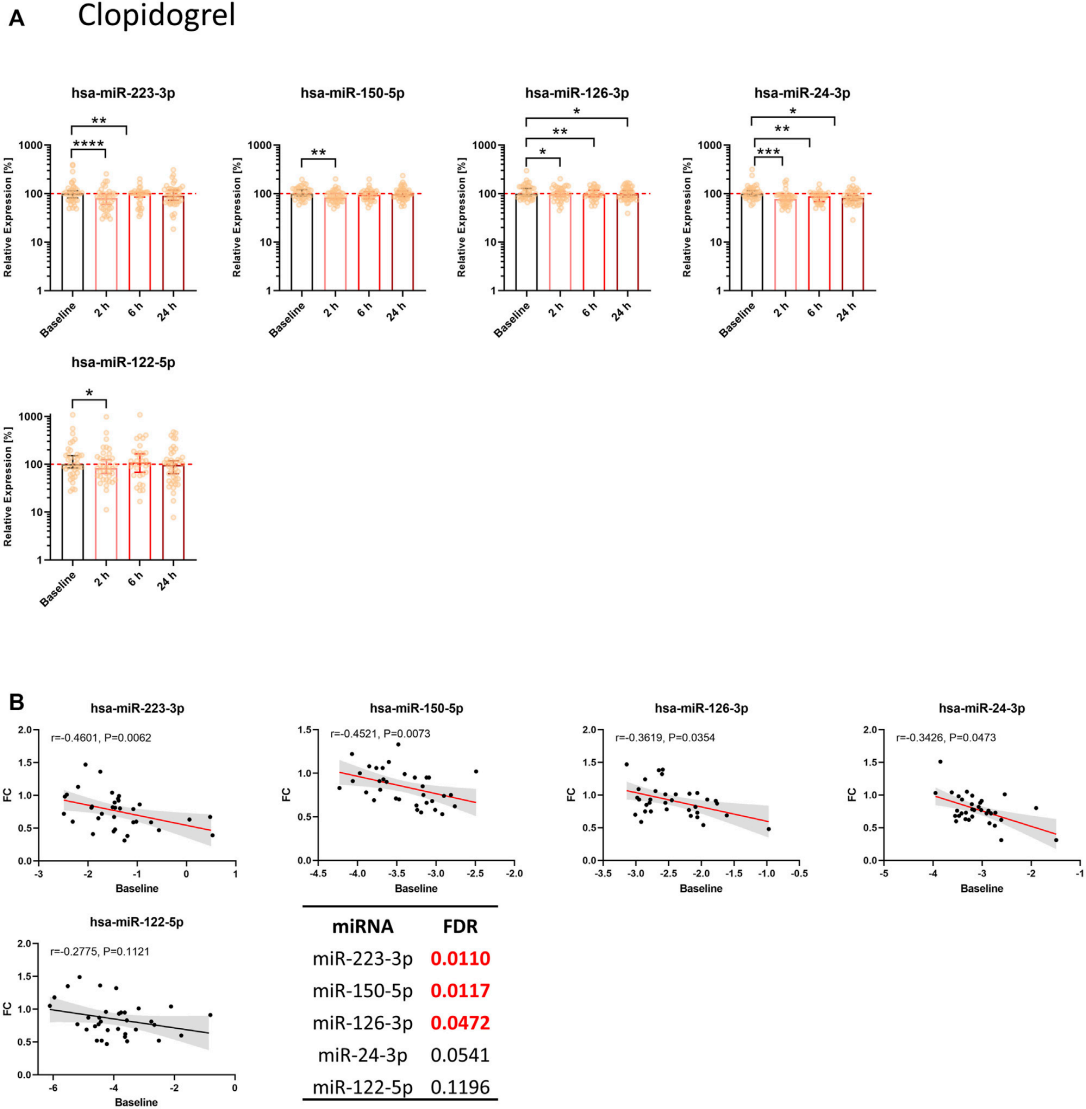
Circulating levels of miRNAs from the thrombomiR panel are significantly reduced upon clopidogrel-mediated inhibition of platelet function. **(A)** MiRNA levels in subjects treated with clopidogrel. Values represent the relative expression of UniSp4 normalized Cq values that were linearized (ΔΔCq, % of the median of baseline samples). Friedman test with Dunn’s multiple comparisons test was calculated. Donors with missing values were removed from statistical analysis but not from depiction in graphs. **(B)** Baseline miRNA levels were correlated with the FC of the decrease 2 h after onset of clopidogrel treatment. Correlations were calculated using the Spearman test, reporting the Spearman rank coefficient (r) and unadjusted *p*-value (P). A line was fit to the data applying simple linear regression. Multiple testing adjusted *p*-values are reported as FDR for each miRNA. FDR-values <0.05 were considered statistically significant.

Clopidogrel significantly reduced the levels of miR-223-3p, miR-150-5p, miR-126-3p, and miR-24-3p (Figure 3A). We observed a decrease compared to the median of baseline samples between ∼1% (miR-126-3p) and ∼22% (miR-24-3p) (Table 1B). MiR-126-3p and miR-24-3p were still significantly decreased after 24 h. Besides the four top candidates, miR-197-3p, miR-27b-3p, and miR-23a-3p were significantly reduced upon clopidogrel treatment as well (Supplementary Figure S3B). Baseline levels of miR-223-3p, miR-150-5p, miR-126-3p, and miR-24-3p correlated with the extent of reduction of circulating miRNA levels 2 h after clopidogrel intake, thus indicating that high baseline levels of circulating miRNAs result in a more pronounced reduction by clopidogrel (Figure 3B). This effect was further investigated by separately analyzing subjects with particularly high miRNA levels and subjects with particularly low miRNA levels. All selected miRNAs (miR-223-3p, miR-150-5p, miR-126-3p, miR-24-3p) were more strongly reduced after 2 h in the 10 donors with the highest baseline levels compared to the donors with the lowest baseline levels (Table 2B). The strongest difference between volunteers with low and high baseline levels was found in miR-24-3p, with a reduction of on average 14% after 2 h compared to 40% in probands with high baseline levels. There was a slight but significant reduction of miR-122-5p levels after 2 h, however the difference between donors with low and high baseline levels was less pronounced compared to platelet-enriched miRNAs and no correlation between miR-122-5p baseline levels and the FC after 2 h was observed.

### 3.4 Association between miRNAs and EVs in subjects treated with clopidogrel

**FIGURE 4:**
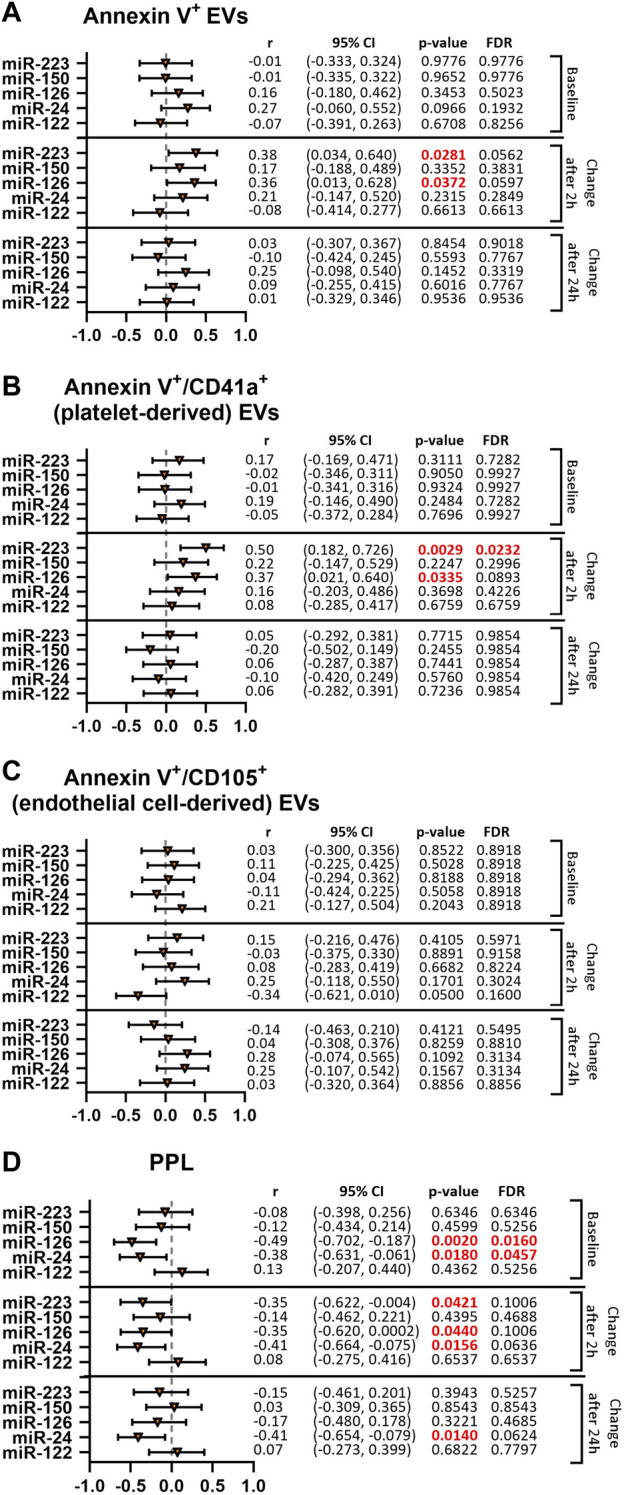
ThrombomiRs show a positive correlation with Annexin V^+^ and platelet-derived (CD41a^+^) EVs as well as a negative correlation with clotting time dependent on PPL upon treatment with clopidogrel. Correlations of thrombomiRs with Annexin V^+^ EVs **(A)**, Annexin V^+^/CD41a^+^ EVs **(B)**, Annexin V^+^/CD105^+^ EVs **(C)**, and clotting time dependent on PPL **(D)**. The baseline (top panel), the change after 2 h (middle panel) or the change after 24 h (bottom panel) were included in the analysis and Spearman’s rank correlation coefficient (r) and the 95% CI are depicted. Multiple testing adjusted *p*-values are reported as FDR for each miRNA. FDR-values <0.05 were considered significant.

We then aimed to better understand the observed reduction of thrombomiR levels upon platelet inhibition. For this purpose, we performed correlation analyses of thrombomiRs with Annexin V^+^, platelet-derived (Annexin V^+^/CD41a^+^), and endothelial cell-derived (Annexin V^+^/CD105^+^) EVs (Figures 4A–C). We report the results including the baseline values only (top panel) and the change after 2 (middle panel), and 24 h (bottom panel). Correlations with the baseline values signify an inherent feature of the cohort that is not induced by P2Y12-mediated platelet inhibition. Correlations between the change in miRNAs and EV concentrations 2 and 24 h after treatment onset illustrate the effect induced by antiplatelet therapy. Baseline levels of thrombomiRs were not correlated with the total number of EVs (Figure 4A). We observed a positive correlation of the change after 2 h of antiplatelet therapy onset between the number of EVs and miR-223-3p (r = 0.38) as well as miR-126-3p (r = 0.36). This effect was not present anymore when correlating the number of EVs with the change in thrombomiR levels 24 h after therapy start. MiR-223-3p (r = 0.5) and miR-126-3p (r = 0.37) were positively correlated with PdEVs 2 h after therapy start. This effect was not present at baseline or when regarding the change after 24 h (Figure 4B). ThrombomiRs from our selection do not seem to be incorporated into endothelial cell-derived EVs as we did not find positive correlations at baseline or as response to platelet inhibition (Figure 4C). Levels of the liver-specific miR-122-5p did not correlate with the number of EVs (independent of the origin) at baseline, 2 or 24 h after antiplatelet treatment.

### 3.5 Association between miRNAs and PPL in subjects treated with clopidogrel

We correlated thrombomiR levels with procoagulant phospholipid activity (PPL) in PPP. This assay measures the clotting time triggered by procoagulant EVs. The more procoagulant EVs are present (increased procoagulant activity), the shorter the coagulation time becomes. At baseline, miR-126-3p (r = −0.49) and miR-24-3p (r = −0.38) were significantly correlated with the clotting time induced by PPL (Figure 4D). The change 2 h after initiation of clopidogrel treatment was negatively correlated with miR-223-3p (r = −0.35), miR-126-3p (r = −0.35), and miR-24-3p (r = −0.41) but not with miR-150-5p. Regarding the change after 24 h, only miR-24-3p (r = −0.41) was still correlated with the clotting time triggered by PPL. For miR-126-3p and miR-24-3p, the correlation seems to be an inherent feature (present at baseline) but is also promoted by pharmacological platelet inhibition. MiR-223-3p was not linked to clotting time dependent on PPL at baseline but correlated after 2 h, indicating an effect of antiplatelet therapy. As expected, levels of miR-122-5p did not correlate with the clotting time induced by PPL at baseline or after therapy start.

## 4 Discussion

We show that platelet-enriched miRNAs in the circulation are significantly reduced upon P2Y12-mediated platelet inhibition. This is in line with previous observations demonstrating that pharmacological inhibition of platelet function is linked to decreased levels of some plasma miRNAs (de Boer et al., 2013; Willeit et al., 2013; Carino et al., 2016; Parker et al., 2020).

However, the associated temporal change of platelet-enriched miRNA levels in plasma remains largely unexplored. We therefore examined the kinetics of miRNA alterations as consequence of platelet inhibition in a panel of miRNAs previously shown to be platelet-enriched and altered upon changes in platelet function (Willeit et al., 2013; Sunderland et al., 2017; Krammer et al., 2022). We found significantly reduced levels of thrombomiRs 2 h after antiplatelet therapy initiation. In total, seven out of eleven thrombomiRs (i.e., miR-223-3p, miR-197-3p, miR-150-5p, miR-126-3p, miR-27b-3p, miR-24-3p, miR-23a-3p) were significantly downregulated in both study cohorts. We found no significant effect of antiplatelet treatment on plasma levels of miR-320a, miR-191-5p, and miR-28-3p. Platelet miRNAs differ in regard to their responsiveness to alterations in platelet function. Highly abundant platelet miRNAs are not necessarily released more readily compared to miRNAs with fewer copies. Therefore, not all thrombomiRs are equally suitable biomarker candidates. Liver-specific miR-122-5p served as negative control (Chang et al., 2004; Willeit et al., 2016). In the ticagrelor cohort, we observed an average decrease of ∼50% after 2 h in miR-223-3p. In the clopidogrel cohort, miR-223-3p levels were decreased by ∼20% (on average). Importantly, the decrease of extracellular miRNAs upon therapy onset occurred fast, reaching its peak after 2 h. In the ticagrelor cohort, the effect lasted for 6 h and rebounded to baseline levels after 24 h. In the clopidogrel cohort, we still observed a significant reduction of miR-126-3p, miR-24-3p, miR-27b-3p, and miR-23a-3p 1 day after treatment initiation. To our knowledge, the rapid kinetics of plasma miRNA decline following pharmacological platelet inhibition have never been shown before.

Our finding is in concordance with several previous studies reporting lower miRNA levels in blood upon (more potent) platelet inhibition (de Boer et al., 2013; Willeit et al., 2013; Carino et al., 2016; Parker et al., 2020). MiR-191-5p is mentioned as potential biomarker candidate in several studies (de Boer et al., 2013; Willeit et al., 2013; Carino et al., 2016; Parker et al., 2020), however, we did not observe a significant response to antiplatelet treatment in healthy volunteers. The effect size of different platelet-related miRNAs in this context varies from study to study, potentially due to different study designs and subjects. For instance, Parker and colleagues found no difference in plasma miR-126-3p levels between donors with type 2 diabetes treated with aspirin or the more potent drug prasugrel (Parker et al., 2020). Overall, the miRNAs previously shown to be responsive to platelet inhibition match our selection of top candidates (de Boer et al., 2013; Willeit et al., 2013; Carino et al., 2016; Parker et al., 2020). One study, however, contradicts our results (Jäger et al., 2019). Plasma levels of platelet-related miRNAs were measured in patients on chronic DAPT at baseline and after cessation of antiplatelet therapy (Jäger et al., 2019). The authors did not detect a rise after therapy discontinuation (after 10, 30, and 180 days) (Jäger et al., 2019). We speculate that miRNA levels might adapt to long-term P2Y12 receptor inhibition (f.i., by inducing transcriptional changes in megakaryocytes), rendering them poor biomarkers for long-term treatment monitoring.

Several mechanisms might contribute to alterations of thrombomiR levels measured in plasma: i.) reduced secretion from platelets (or megakaryocytes) due to platelet inhibition; ii.) increased degradation or uptake; iii.) altered transcription in megakaryocytes; iv.) altered maturation of pre-miRNAs in platelets. The half-life period of plasma miRNAs from our panel is not known. In case the half life is short, an inhibition of the secretion could cause the observed effect. The exact mechanisms need to be elucidated in future studies.

MiRNAs from our panel are also detectable at baseline as they are enriched in platelets but not platelet-specific. Active or passive release from other cell types likely contributes to basal levels. MiR-223-3p and miR-150-5p are also expressed in leukocytes (de Rie et al., 2017; Krammer et al., 2022). MiR-126-3p is abundant in endothelial cells, while miR-24-3p shows broad expression among different tissues (de Rie et al., 2017). Nevertheless, platelets substantially contribute to the pool of cell-free miRNAs in blood (Willeit et al., 2013; Kaudewitz et al., 2016). Even though it has been suggested that platelets mainly release miRNAs upon activation, some degree of passive leakage cannot be ruled out.

In accordance with previous studies that have reported no substantial difference between MAPT and DAPT with regard to parameters of hemostatic system activation in healthy volunteers (Traby et al., 2021, Traby et al., 2020), miRNAs from our panel were not influenced by the addition of aspirin.

Additionally, we observed that the effect size (fold reduction) of miRNAs was dependent on baseline miRNA levels. Therefore, we speculate that effect sizes might be different in CVD patients with potentially altered baseline levels.

To gain more insight into the origin of the detected miRNA signal, we correlated thrombomiR levels with previously obtained EV concentrations (Traby et al., 2018). The number of EVs exposing platelet markers are increased in thrombotic diseases and positively correlated with disease activity (van der Zee et al., 2006; Bal et al., 2010; Stępień et al., 2012; Giannopoulos et al., 2014; Chen et al., 2015). The main carrier of extracellular miRNAs remains a matter of controversy and likely differs between miRNAs. We previously reported that basal levels of platelet-related miRNAs are mainly protein-bound (Krammer et al., 2022). Activated platelets have been shown to secrete EVs with miRNAs as cargo (Diehl et al., 2012; Gidlöf et al., 2013; Laffont et al., 2013). It is currently unclear whether quiescent platelets also secrete EVs *in vivo*. Our results indicate that thrombomiRs might circulate the body within EVs and that this association is not an innate feature but induced by alterations of platelet function. Especially the change after 2 h of miR-223-3p and miR-126-3p was robustly correlated with the number of PdEVs. MiRNAs from our selection were not significantly correlated with endothelial cell-derived (CD105^+^) EVs. This was also true for miR-126-3p that is also expressed in the endothelium (Zampetaki et al., 2012a; Kaudewitz et al., 2016; de Rie et al., 2017; Sunderland et al., 2017). Moreover, thrombomiRs (change after 2 h of clopidogrel treatment) were negatively correlated with clotting time dependent on procoagulant phospholipids (PPL assay), indicating that treatment with P2Y12 inhibitors affects the amount of procoagulant EVs carrying platelet miRNAs. Our study design does not permit to determine whether thrombomiRs in procoagulant EVs are merely an indicator of the cellular source or have functional implications. Procoagulant EVs may contribute to hypercoagulability and enhance thrombogenesis in patients (Nielsen et al., 2018). It has been proposed that platelets and megakaryocytes substantially contribute to the pool of circulatory EVs (Edelstein, 2017; Stone et al., 2022). As of now, it is technically not feasible to distinguish between EVs released from platelets or megakaryocytes. Despite promising results, the EV field is still in its infancy. The miRNA cargo of PdEVs needs to be sufficiently characterized. This endeavour is presently limited by technological pitfalls involving the reproducible extraction and detection of EVs. Also, the biological relevance of nucleic acids found within EVs remains controversial. It is unclear whether PdEVs play an active role in coagulation.

The present work is subject to specific strengths and limitations. Quantification of extracellular miRNAs is frequently confounded by improper blood sample handling, including cellular contamination. To avoid this common pitfall, we measured our miRNA panel in double-centrifuged PPP with negligible amounts of residual platelets. Only miR-126-3p in the clopidogrel collective was mildly correlated with platelet count, indicating that our data set is not biased in this regard. Blood samples were anticoagulated with sodium citrate. To avoid artificial platelet activation when studying platelet-derived molecules in plasma, the use of citrate-containing anticoagulants is recommended (Mussbacher et al., 2020, 2017). We only included healthy volunteers and no patients with CVD in this study. This approach allowed us to work with a well-characterized and homogenous cohort. In addition, only men were included to avoid biasing results by factors other than drug intake. Our experimental protocols did not allow us to discriminate between different miRNA fractions in blood (protein vs EV), as we extracted total RNA from plasma, which produces a composite signal of miRNAs bound to proteins or complexed with EVs. We did not directly determine the cellular source of plasma thrombomiRs but performed correlation analyses with previously collected data. Further disadvantages of the selected study design are the resulting lack of clinical endpoints and the unavailability of long-term data.

## 5 Conclusion

At the moment, routine assessment of platelet function is not recommended in the course of antiplatelet treatment (Collet et al., 2021). Some studies, however, report a potential benefit (Stone et al., 2013; Aradi et al., 2015; Sibbing et al., 2017). Our work provides evidence that platelet-enriched miRNAs in the circulation are promising biomarker candidates for monitoring the extent of platelet inhibition. This could enable the stratification of patients depending on their response to antiplatelet therapy and guide treatment decisions. For the first time, we demonstrate the rapid effect of P2Y12 receptor inhibition on miRNA levels. Follow-up studies in patient cohorts to examine the impact of increased levels of platelet activation, age, and co-morbidities are warranted. Our evidence suggests a connection between platelet activation/inhibition and the levels of thrombomiRs in plasma that could be exploited for antiplatelet drug development as well as in clinical practice.

## Data Availability

The raw data supporting the conclusions of this article will be made available by the authors without undue reservation.
