# Supplementary material for: Plasma levels of platelet-enriched microRNAs change during antiplatelet therapy in healthy subjects
Source: Front Pharmacol. 2022 Dec 12;13:1078722. doi: 10.3389/fphar.2022.1078722 (PMC9790905; doi:10.3389/fphar.2022.1078722)
Supplement: Supplementary file 1 [file Presentation1.pdf]

Supplementary Figures

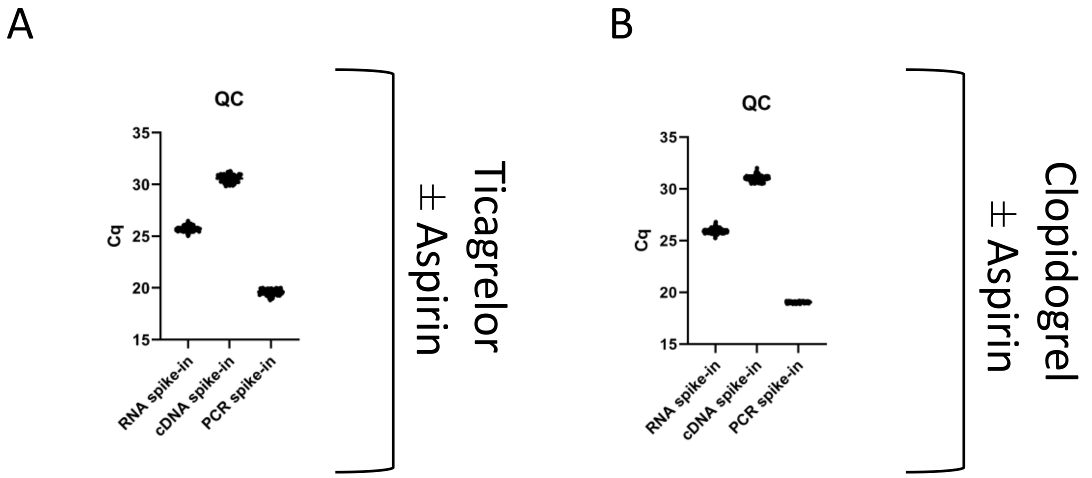

Supplementary Figure 1. Quality control using synthetic RNA spike-ins added in equimolar amounts before RNA extraction, reverse transcription, and qPCR.

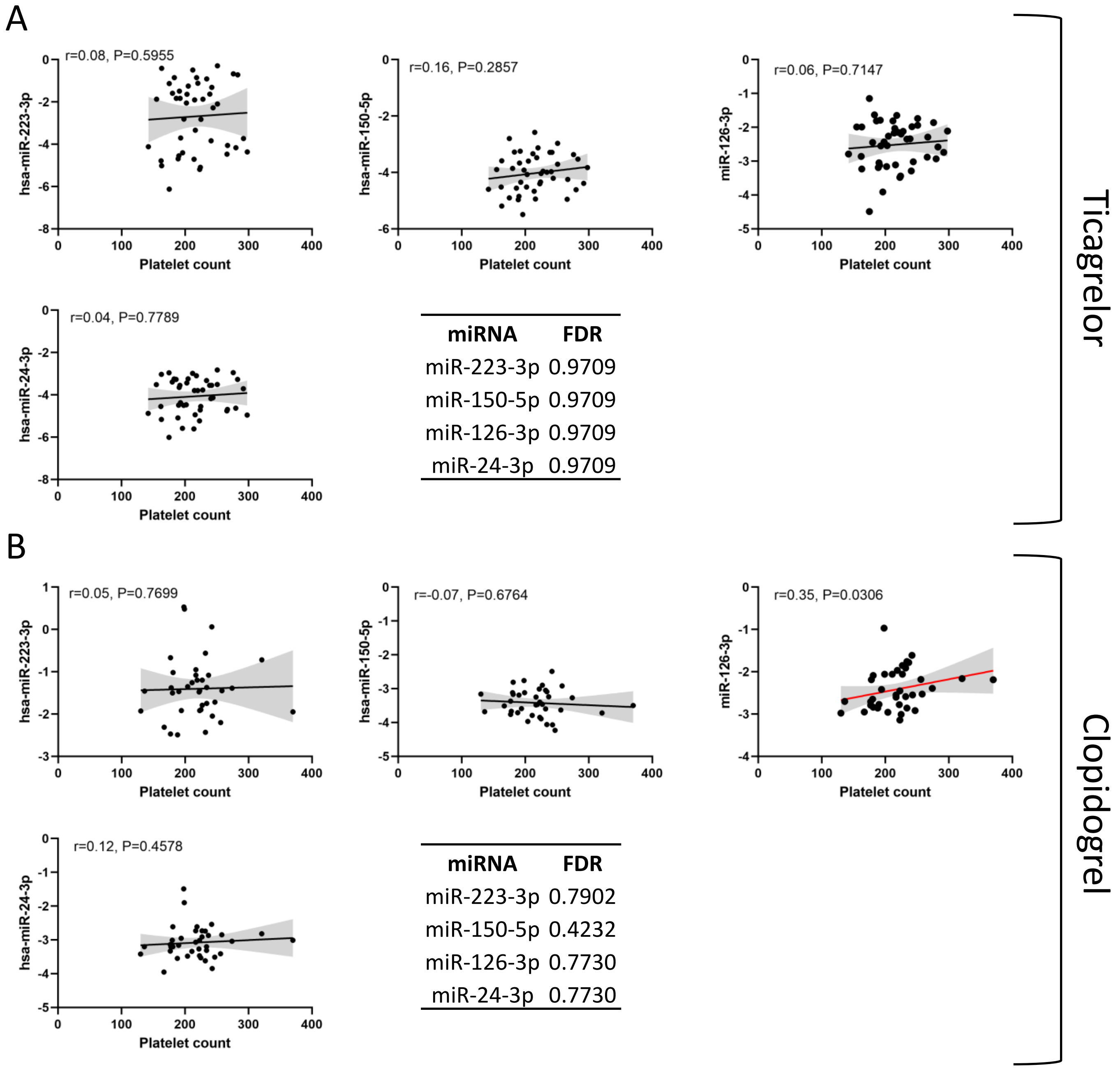

miRNA	FDR
miR-223-3p	0.9709
miR-150-5p	0.9709
miR-126-3p	0.9709
miR-24-3p	0.9709

miRNA	FDR
miR-223-3p	0.7902
miR-150-5p	0.4232
miR-126-3p	0.7730
miR-24-3p	0.7730

Supplementary Figure 2. Circulating thrombomiR levels are not correlated with platelet counts at baseline, indicating that the preparation of PPP by double centrifugation was successful in removing residual platelets.

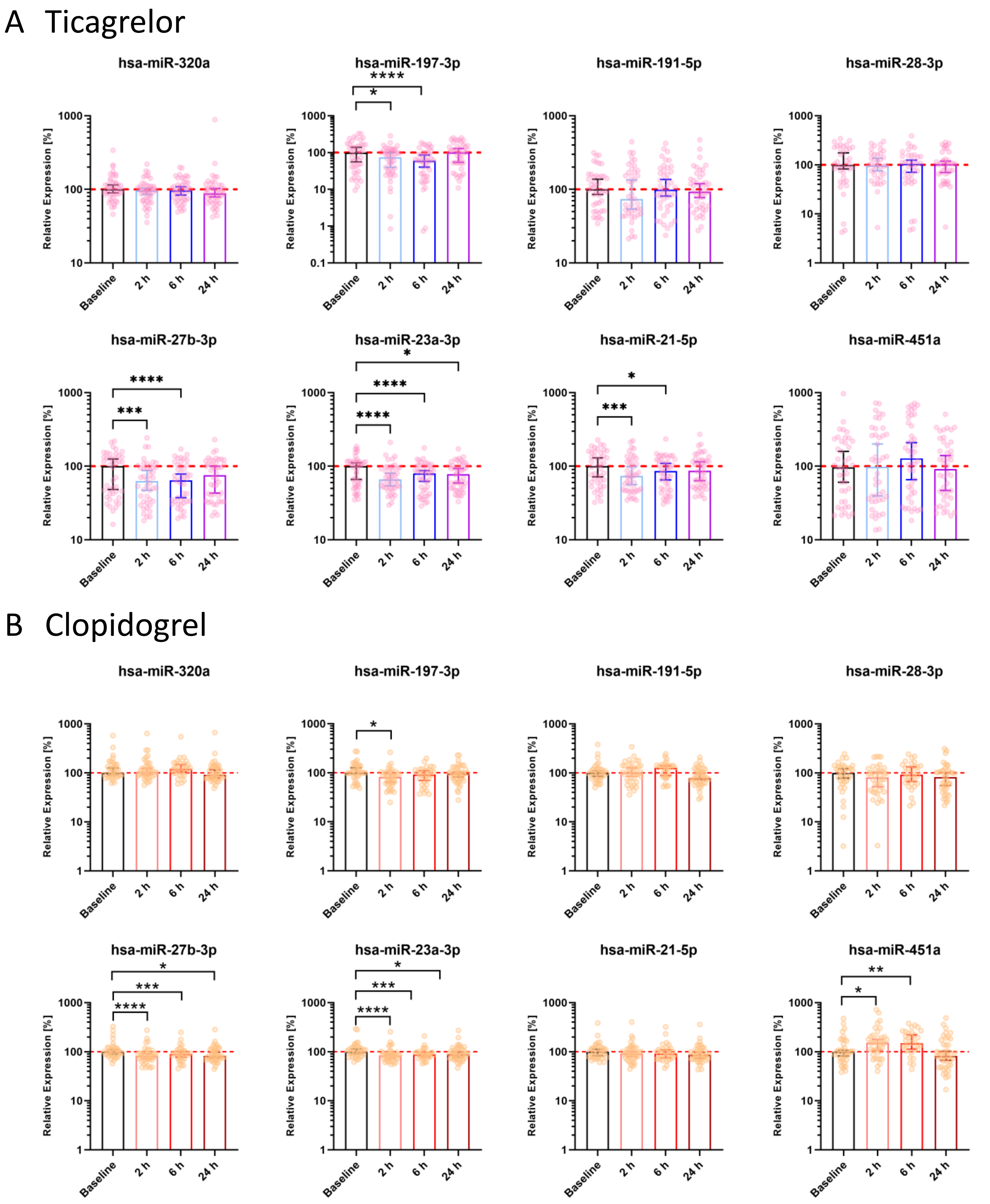

Supplementary Figure 3. ThrombomiR levels in the course of P2Y12-mediated antiplatelet treatment: (A) MiRNA levels in subjects receiving ticagrelor treatment; (B) miRNA levels in subjects treated with clopidogrel. Values represent relative expression of UniSp4 normalized Cq values that were linearized (ΔΔCq, % of the median of baseline samples). Friedman test with Dunn's multiple comparisons test was calculated. Donors with missing values were removed from statistical analysis but are depicted in the graphs. P-values <0.05 were considered significant.
